# Mechanisms by which HPV Induces a Replication Competent Environment in Differentiating Keratinocytes

**DOI:** 10.3390/v9090261

**Published:** 2017-09-19

**Authors:** Cary A. Moody

**Affiliations:** 1 Department of Microbiology and Immunology, University of North Carolina at Chapel Hill, Chapel Hill, NC 27599, USA; camoody@med.unc.edu; 2 Lineberger Comprehensive Cancer Center, University of North Carolina at Chapel Hill, Chapel Hill, NC 27599, USA

**Keywords:** virus, HPV, cell cycle, differentiation, replication, DNA damage response

## Abstract

Human papillomaviruses (HPV) are the causative agents of cervical cancer and are also associated with other genital malignancies, as well as an increasing number of head and neck cancers. HPVs have evolved their life cycle to contend with the different cell states found in the stratified epithelium. Initial infection and viral genome maintenance occurs in the proliferating basal cells of the stratified epithelium, where cellular replication machinery is abundant. However, the productive phase of the viral life cycle, including productive replication, late gene expression and virion production, occurs upon epithelial differentiation, in cells that normally exit the cell cycle. This review outlines how HPV interfaces with specific cellular signaling pathways and factors to provide a replication-competent environment in differentiating cells.

## 1. Introduction

Human papillomaviruses (HPV) are non-enveloped, small DNA viruses that exhibit a strict tropism for epithelial cells. Over 200 types of HPVs have been identified and are classified into five evolutionary genera (α, β, γ, μ, v) based on DNA sequence similarity [1]. The alpha group is the largest, containing approximately 64 HPV types, and is divided based on tropism of each type for cutaneous or mucosal epithelium. The cutaneous types cause common warts, which are rarely associated with malignancy [2]. About 40 alpha HPVs infect the mucosal epithelium and are categorized as high-risk or low-risk based on their association with cancer [1,3]. Low-risk types (e.g., HPV11 and HPV6) are most commonly associated with benign genital warts, but are also implicated in the development of laryngeal papillomas. The fifteen types termed high-risk (16, 18, 31, 33, 35, 39, 45, 51, 51, 56, 58, 59, 68, 73, 82) are classified as oncogenic based on their association with anogenital cancers [4]. In addition, certain high-risk types, particularly HPV16, infect the oropharyngeal mucosa and are associated with an increasing number of head and neck cancers [5]. Beta HPVs exhibit a tropism for the cutaneous epithelium, with infection occurring early in life and typically producing an asymptomatic infection [6]. However, persistent infection with certain types of beta HPVs are associated with the development of non-melanoma skin cancers at sun exposed sites, particularly in immunosuppressed patients and patients with the rare disease epidermodysplasia verruciformis [7]. The mu, nu and gamma HPVs infect the cutaneous epithelium and are most commonly associated with the formation of benign papillomas [8].

## 2. HPV Life Cycle

**Figure 1:**
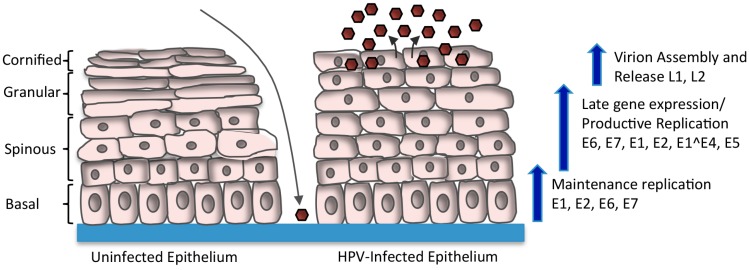
Human papillomavirus (HPV) Life Cycle. HPV infects the basal layer of the stratified epithelium through a microwound. Upon entry into the cell, the virus transiently amplifies to 50–100 copies per cell. HPV genomes are maintained at a stable copy number in undifferentiated basal cells by replicating along with cellular DNA. Upon differentiation, the productive phase of the life cycle is activated, resulting in late gene expression and amplification of viral genomes to thousands of copies per cell. The expression of E6 and E7 allows for cell cycle re-entry upon differentiation, providing cellular factors for productive replication. E4 and E5 also contribute to efficient productive replication. Expression of L1 and L2 promotes the encapsidation of newly replicated genomes, resulting in virion release from the uppermost layers of the epithelium (brown hexagons).

The life cycle of HPV is intimately linked to the differentiation status of the host cell keratinocyte and is characterized by three distinct phases of replication [9,10] (Figure 1). High-risk and low-risk HPVs initiate infection by gaining access to the proliferating basal cells of the stratified epithelium through a microwound [11]. Upon entry, HPV undergoes a transient round of replication referred to as “establishment replication”, which results in a copy number of 50–100 viral genomes per cell. Viral episomes are subsequently maintained in the undifferentiated basal cells by replicating along with the host cell chromosomes. Only upon epithelial differentiation is the productive phase of the viral life cycle activated, resulting in the amplification of viral genomes to thousands of viral copies per cell in the suprabasal layers, as well as activation of late gene expression and virion assembly and release [10,12]. Regulation of the viral life cycle in this manner allows HPV to avoid detection by the immune response as high levels of viral gene expression as well as virion production are restricted to the uppermost layers of the epithelium, which are not under immune surveillance [4].

Due to the small coding capacity of the viral genome, HPV depends on the host DNA replication machinery to synthesize its DNA. While readily available in undifferentiated cells to stably maintain viral episomes, epithelial differentiation normally results in an exit from the cell cycle, limiting the availability of replication machinery in post-mitotic cells [13]. This provides a conundrum for HPV since differentiation is required to activate the productive phase of the life cycle, yet HPV also depends on cellular factors for replication. To support productive replication, HPV employs numerous mechanisms to subvert key regulatory pathways that regulate host cell replication, in turn maintaining differentiating cells active in the cell cycle. As such, HPV is able to reactivate cellular genes and signaling pathways necessary to support late gene expression and amplification of viral DNA. The majority of our insights into productive HPV replication have emerged from studying the alpha HPV types, primarily the high-risk types HPV16, HPV18 and HPV31. This review will focus on the mechanisms by which alpha HPVs renders post-mitotic, differentiating cells permissive for DNA synthesis during the productive phase of the viral life cycle.

## 3. HPV Genome Organization

**Figure 2:**
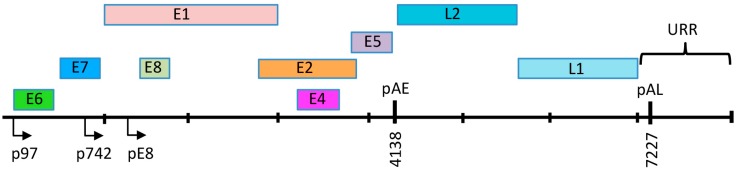
Linear depiction of the HPV31 genome. The open reading frames (ORF) are indicated by the color blocks. The early promoter is located upstream of the E6 ORF (p97) and the late promoter is located in the E7 ORF (p742). E8^E2 is expressed from a promoter located in the E1 ORF (pE8). The early polyadenylation site is located at the 3’ end of the E5 ORF (pAE) and the late polyadenylation site (pAL) is located in the URR (Upstream Regulatory Region). The origin of replication, as well as E1 and E2 binding sites are also located in the URR.

The HPV genome exists as a covalently closed circle (episome) of approximately 8 kb [8]. HPV genomes are histone-associated in the virion as well as in infected cells, exhibiting a nucleosomal pattern similar to that of cellular DNA [14,15]. HPV genomes contain six to eight open reading frames (ORF) that are expressed as polycistronic transcripts that are then alternatively spliced to yield individual gene products [16,17] (Figure 2). High-risk HPV genomes contain two main promoters that are active at different stages in the viral life cycle [18,19,20]. In undifferentiated epithelial cells, viral gene expression is regulated by the early promoter, which is located adjacent to the E6 ORF in the upstream regulatory region (URR) and is referred to as p97 for HPV16 and HPV31, and p105 for HPV18. The early promoter directs expression of E1 and E2, which is necessary for viral replication. E1 is an ATP-dependent helicase that facilitates unwinding of the viral DNA and also recruits cellular factors to the viral origin of replication, located in the URR [21]. E2 is a sequence-specific DNA binding protein that has multiple binding sites in the URR. E2 binds and recruits E1 to a specific E1 binding site in the viral origin. E2 also regulates viral gene expression from the early promoter. In addition, E2 contributes to episomal maintenance in undifferentiated cells by tethering viral genomes to host mitotic chromosomes [22]. E6 and E7, which are the oncoproteins for the high-risk HPV types, are also expressed from the early promoter. E6 and E7 contribute to viral replication through their ability to modulate cell cycle control, cell survival, cellular differentiation, immune evasion, as well as DNA damage responses [23,24,25,26,27]. E1^E4 is encoded by a spliced RNA that fuses the first five amino acids of the E1 ORF with E4 [28]. While E1^E4 and E5 are expressed at low levels from the early promoter in undifferentiated cells, the high-risk E4 and E5 proteins seem to be primarily involved in facilitating efficient productive replication in differentiating cells [29,30,31,32,33]. Some HPV types express a fusion of E8 and the C-terminal half of the E2 ORF (E8^E2), which initiates from a promoter in the E1 ORF and functions to limit viral replication and transcription in undifferentiated and differentiated cells [34]. The late promoter is located in the E7 ORF (p742 HPV31, p811 HPV18, p670 HPV16) and is activated upon epithelial differentiation [35,36,37]. The late promoter is not regulated by E2 and drives high levels of expression of E1 and E2, as well as E1^E4 and E5 to facilitate productive viral replication. In addition, the late promoter directs expression of the L1 and L2 capsid genes to allow for encapsidation of viral genomes in the uppermost layers of the epithelium.

## 4. Regulation of Viral Gene Expression upon Keratinocyte Differentiation

Efficient amplification of HPV genomes upon differentiation requires activation of the late promoter to provide increased levels of E1, E2, E1^E4 and E5 [10]. The early promoter remains active upon differentiation, directing expression of E6 and E7, which is also necessary for late viral events. The tight link between differentiation and late gene expression suggests that differentiation-specific factors are required for late promoter activation. Chromatin rearrangements and histone modifications are detected at the late promoter upon differentiation, though how this is regulated remains unclear [38,39]. A variety of transcription factors have been shown to bind to the late promoter in the context of complete, episomal genomes, both in undifferentiated and differentiated cells, including c-Myb, C/EBPα, C/EBPβ, NFAT (Nuclear Factor of Activated T-cells), YY1 (Yin Yang 1), NF1 (Nuclear Factor 1), Oct-1 (Octamer-binding transcription factor 1), c-Jun, and Sp1 (Specificity Protein 1) [38,40]. However, only the LIP (Liver-enriched Inhibitory Protein) and LAP (Liver-enriched Activator Protein) isoforms of C/EBPα have been shown to regulate late promoter activity [41]. More recent studies have shown that transcription elongation regulates late promoter activity through the recruitment of elongation mediators (e.g., CDK8, BRD4) to viral genomes upon differentiation [42]. Late gene expression is also regulated by alternative splicing and changes in polyadenylation site usage [16]. Upon differentiation, read-through of the early polyadenylation site (pAE) located at the end of the E5 ORF allows late transcripts to be polyadenylated at the late polyadenylation (pAL) site located in the URR, facilitating expression of L1 and L2. Transcriptional read-through may be influenced by E2 expression, which increases in the mid to upper epithelial layers of the epithelium and has been shown to repress polyadenylation at the early site [43,44,45]. Polyadenylation is co-transcriptionally regulated with splicing, and certain splicing factors have been shown to influence polyadenylation site usage for HPV16 [46,47]. Splicing of HPV transcripts is positively regulated by Serine Arginine splicing factors (SRSF) (e.g., SRSF1, SRSF2, SRSF3), which increase upon differentiation and are regulated transcriptionally by E2 [46,48]. SRSF9 has also been shown to increase the efficiency of late RNA splicing [49]. Studies by the Parish lab recently demonstrated that CTCF insulator proteins regulate viral transcript splicing upon differentiation through binding to the HPV18 E2 ORF [50]. Mutation of the E2 CTCF binding site in the context of the HPV18 genome results in increased levels of E6 and E7 and increased proliferative capacity in suprabasal cells [50]. The E2 CTCF binding site is conserved across high-risk types, suggesting that HPV has evolved CTCF recruitment to viral genomes to control the levels of E6 and E7 upon differentiation, possibly to facilitate eventual exit from the cell cycle to allow for virion assembly and release.

## 5. Maintenance of Proliferative Potential in Differentiating Cells

### 5.1. Disruption of Rb/E2F Complexes

**Figure 3:**
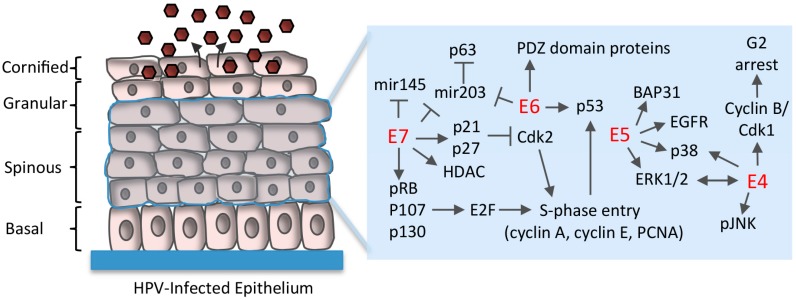
Cellular factors targeted by HPV proteins to maintain cell cycle competency in suprabasal cells. E7 pushes differentiating cells back into the cell cycle by binding to pRb, p107 and p130, which regulate entry into S-phase by negatively regulating E2F transcription factors. Disruption of the pRb/E2F interaction by E7 allows for constitutive activation of E2F-responsive genes, allowing for S-phase re-entry by post-mitotic cells. Unscheduled S-phase entry induced by E7 results in increased p53 that is targeted for degradation by E6 to avoid apoptosis or cell cycle arrest in G1, as well as to block p53’s negative effects on productive replication. E5 contributes to productive viral replication by maintaining cell cycle competency upon differentiation through interaction with BAP31, as well as through activation of epidermal growth factor receptor (EGFR), mitogen activated protein kinase (p38MAPK) and extracellular signal-regulated kinase (ERK)1/2. E4 may increase the efficiency of viral genome amplification by sustaining a G2-arrested environment upon differentiation, and through activation of MAPK signaling (p38, ERK1/2, pJNK). T bars indicate inhibition. Arrows indicate activation. HDAC: histone deacetylase; PCNA: proliferating cell nuclear antigen.

As normal, uninfected cells leave the basal layer, they lose proliferative potential and begin a terminal differentiation program [13]. However, a fundamentally important aspect of the HPV life cycle is to maintain cell cycle competence in differentiating epithelial cells to provide cellular factors for productive replication. E7 plays a critical role in this process though the binding and targeted degradation of the tumor suppressor pRb, as well as the related pocket proteins p107 and p130 [51] (Figure 3). Rb family members regulate the G1 to S-phase transition by controlling the activity of E2F transcription factors [52]. E7 binds to Rb family members through a conserved LXCXE domain located in the extreme C-terminus that disrupts the interaction between Rb and E2F transcription factors [53,54]. Disruption of the Rb/E2F interaction results in constitutive activation of E2F-resposive genes, allowing E7 to push differentiating cells back into S-phase, disrupting suprabasal quiescence and reactivating cellular DNA synthesis [55,56,57,58]. As a result, suprabasal cells exhibit markers of differentiation, as well as markers of cell cycle re-entry, including PCNA, cyclin A and cyclin E [55,59]. Low-risk E7 proteins also bind pRb, p107 and p130, but with much lower affinity, and only target p130 for degradation [53,60,61]. The loss of E7 expression in the context of HPV16 infection prevents the induction of host cell replication machinery and productive viral replication in suprabasal epithelial cells of organotypic raft cultures, which recapitulate the three-dimensional architecture of the stratified epithelium [62,63]. These studies highlight the importance of E7 in differentiation-dependent viral events. In addition to Rb family members, the interaction between E7 and type 1 histone deacetylases (HDAC1-3) is also important in maintaining E2F activation upon differentiation and facilitating viral replication [64,65,66]. HPV31 E7 specifically increases the levels of E2F2 by preventing HDAC binding to the *e2f2* promoter [65]. The increase in E2F2 is necessary for productive viral replication, though the downstream targets of E2F2 have not yet been identified.

### 5.2. Uncoupling of Differentiation From Proliferation

Normal epithelial differentiation results in cell cycle arrest that is carried out by increased expression of the cyclin-dependent kinase inhibitors p21Cip1 and p27Kip1, which inhibits the activity of cyclin-dependent kinase 2 (Cdk2) [67]. Cdk2 facilitates G1 to S-phase entry and progression through interaction with cyclin E and cyclin A, respectively [68]. To circumvent this potential block, E7 targets cellular molecules that link differentiation with cell cycle exit (Figure 3). E7 does not affect the differentiation-dependent increase in p21, but rather binds to p21, at least in part through its Rb binding domain, in turn delaying differentiation and blocking the inhibitory effects on Cdk2 activity to establish a proliferative environment [57,69]. Low-risk E7 proteins do not bind as efficiently to p21 and are therefore not as successful at mitigating the inhibitory effects of p21 [57]. High-risk E7 proteins also maintain Cdk2 activity through direct interaction with cyclin E and cyclin A, as well as through maintaining high levels of the Cdc25a phosphatase, which removes inhibitory phosphorylation from Cdk2 [70,71,72]. Cdk2 activity may also contribute to productive viral replication by regulating the cellular localization of the E1 viral helicase. Cdk2-dependent phosphorylation of E1 prevents its nuclear export, leading to accumulation of E1 in the nucleus, which may allow for rapid amplification of viral genomes upon differentiation [73,74]. Proliferative potential in differentiating cells is also maintained by the E5 protein. E5 is expressed at high levels in suprabasal cells and contributes to efficient productive replication of HPV16 and HPV31 [33,75]. Loss of E5 expression in the context of the HPV31 genome results in decreased cyclin A and cyclin B levels upon methylcellulose-induced differentiation and reduces colony formation following differentiation [33]. Colony formation requires E5’s ability to interact with B cell associated protein 31 (BAP31), an ER chaperone and regulator of apoptosis [76]. However, the mechanism by which this interaction maintains proliferative competence in differentiating cells is currently unclear. E5 also modulates several growth pathways that may contribute to viral genome amplification, including signaling through the epidermal growth factor receptor (EGFR), as well as activation of p38MAPK and ERK1/2 [77].

### 5.3. E6 Abrogation of p53 and Targeting of PDZ (PSD95/DLG1/ZO-1) Domain-Containing Proteins

To facilitate re-entry into the cell cycle and viral genome amplification in suprabasal cells, the activities of E7 coordinate with those of E6 [26] (Figure 3). One of the key functions of E6 is the inactivation of p53. For high-risk types, E6 promotes p53 ubiquitylation and proteasome-dependent degradation through interaction with the E6AP ubiquitin ligase [78,79,80]. E6-mediated p53 degradation is thought to protect cells from apoptosis or growth arrest due to E7-mediated cell cycle re-entry in the suprabasal layers. However, recent studies indicate that p53 negatively regulates viral genome amplification. E6 mutants in the context of the HPV18 genome that are unable to destabilize p53 result in fewer suprabasal cells supporting viral genome amplification in organotypic raft cultures [81]. The mechanism by which p53 negatively regulates productive viral genome amplification is unclear, but may be through interaction with the E2 origin binding protein [82,83]. E6 proteins also contribute to replication competence through the targeting of specific cellular proteins containing PDZ (PSD95/DLG1/ZO-1) domains [84]. E6 interacts with PDZ proteins through a C-terminal PDZ domain binding motif (PBM) that is found only in high-risk E6 proteins, suggesting this motif serves as a signature for oncogenic potential [85,86]. Most of the PDZ proteins that interact with E6 are targeted for proteasome-dependent degradation, or have an altered cellular localization [84]. PDZ proteins shown to associate with E6 are involved in the regulation of cell growth and polarity, as well as signal transduction pathways involved in cell proliferation, apoptosis, migration and intracellular trafficking [84]. The E6 PBM has been shown to play an essential role in viral genome amplification. Human foreskin keratinocytes transfected with HPV18 genomes containing a mutation in the E6 PBM exhibit a loss of productive viral replication and late gene expression in organotypic raft cultures, correlating with a decrease in the number of S-phase competent cells in the suprabasal layer [87]. A role for the E6 PBM in productive replication has also been observed for the high-risk types HPV31 and HPV16 [88,89]. More recent studies demonstrated that the E6 PBM protects the mitotic integrity of keratinocytes containing HPV18 episomes, with loss of the PBM domain leading to mitotic abnormalities that prevents the expansion of suprabasal cells to support vegetative viral replication [90]. What specific PDZ proteins are targeted by E6 to preserve mitotic integrity and to promote viral replication have yet to be defined.

### 5.4. Regulation of Differentiation-Induced microRNA Expression

While the HPV genome does not encode microRNAs, E6 and E7 of high-risk types have been shown to modulate the expression of cellular microRNAs to facilitate viral replication in differentiating cells [91] (Figure 3). microRNA-203 (mir203) is normally induced concomitantly with epithelial differentiation and restricts the proliferative potential of differentiating cells by repressing the expression of the p53 homolog p63 [92]. p63 is required for maintaining proliferative potential and acts as a switch between proliferation and differentiation [93]. Studies from the Laimins lab demonstrated that p63 is required for productive replication of HPV31 [94]. Expression of HPV31 E6 and E7 prevents upregulation of mir203 upon differentiation, which is necessary to maintain p63 in differentiating cells and presumably provide a proliferative environment for productive viral replication [95]. In support of this, knockdown of p63 expression in differentiating HPV31 positive keratinocytes using shRNAs results in decreased levels of cell cycle proteins, including cyclins A, B, and E, as well as Cdc25c, Cdk1 and Cdk2 [94]. mir145 is also normally induced upon differentiation and has been shown to negatively regulate the productive phase of the HPV31 life cycle [96]. mir145 regulates the levels of the transcription factor KLF4 (Kruppel-like factor 4), which is a target gene of p63 that plays a role in proliferation, differentiation, and maintenance of stem cells [97,98]. In the stratified epithelium, KLF4 also regulates expression of late epidermal differentiation markers and contributes to the formation of the cornified layer. KLF4 is present at high levels upon differentiation in HPV31 positive cells and is necessary for the productive phase of the viral life cycle [99]. HPV31 regulates KLF4 levels transcriptionally by p63, but also post-transcriptionally by E7-mediated suppression of differentiation-induced mir145 expression [96,99]. KLF4 levels are also regulated post-translationally by E6’s ability to prevent inhibitory phosphorylation and sumoylation of KFL4 [99]. KLF4 directly activates late viral gene expression, and thus productive viral replication, by binding to the HPV31 URR in a complex with BLIMP1. Furthermore, KLF4 expression is necessary to maintain cyclin A and cyclin B in suprabasal cells [99]. KLF4 therefore has multiple functions in promoting the productive phase of the viral life cycle. In addition to KLF4, p63 also regulates expression of the DNA repair factors Rad51 and BRCA2, as well as activation of the checkpoint kinase Chk2 in HPV31 positive keratinocytes [94]. As described in more detail below, Chk2 kinase activity and Rad51 have been shown to be required for productive replication of HPV31 [100,101]. These studies suggest that upon cell cycle re-entry, HPV’s ability to modulate differentiation-induced microRNAs results in maintenance of p63 levels, prolonging proliferative potential and ensuring the expression of a subset of cellular genes necessary for productive viral replication as well as late gene expression. In addition, p63 may contribute to activation of the DNA damage response that is necessary for viral DNA synthesis in differentiating cells.

## 6. Establishment of a G2-Arrested Environment

To provide a replication-competent environment upon differentiation, high-risk and low-risk E7 proteins push post-mitotic cells back into the cell cycle, rather than maintaining cells active in S-phase upon differentiation [102,103]. E7-induced cell cycle re-entry has traditionally been thought to result in an S-phase environment that provides HPV access to replication machinery that supports productive viral replication. However, more recent studies indicate that productive viral replication occurs post-cellular DNA synthesis in cells that are subsequently arrested in G2 [104,105,106]. Using organotypic raft cultures of HPV18 positive keratinocytes, Wang et al., demonstrated that cells undergoing viral genome amplification exhibit markers of G2/M arrest, including high levels of cytoplasmic cyclin B1 and inactive cyclin-dependent kinase 1 (Cdk1) [105]. Cdk1 normally forms a complex with cyclin B1 in the nucleus to stimulate entry into mitosis. In addition, these cells also contain the inactive form of the Cdc25C phosphatase, which functions to remove inhibitory phosphorylation from Cdk1 to allow entry into mitosis [107]. Overall, these studies indicate that HPV requires G2 arrest upon differentiation to support the productive phase of the viral life cycle.

The mechanism by which HPV induces G2 arrest upon differentiation is currently unclear. Arrest in G2 typically occurs in response to DNA damage or incomplete replication, which activates the ATM (Ataxia-Telangiectasia Mutated) and ATR (ATM and Rad3-related) DNA damage kinases [108]. ATM and ATR phosphorylate the checkpoint kinases Chk2 and Chk1, leading to their activation and the phosphorylation/inhibition of Cdc25C, preventing activation of the Cdk1/cyclin B1 complex [109]. As discussed below, high-risk HPV positive cells exhibit constitutive activation of ATM and ATR, with activation of both of these pathways necessary for productive viral replication [100,110,111]. Inhibition of Chk2 kinase activity in differentiating HPV31 positive cells results in decreased inhibitory phosphorylation of Cdc25C and Cdk1, offering support that activation of the ATM/ATR pathways contributes to the G2 arrest observed upon differentiation [100]. E7 expression alone is sufficient to induce ATM and ATR activation, as well as high levels of cytoplasmic cyclin B and Cdk1 in suprabasal cells of HPV18 organotypic raft cultures, suggesting that E7 is involved in facilitating cell cycle arrest upon differentiation [100,104,110]. However, several studies have shown that the overexpression of the E4 protein of multiple HPV types induces G2 arrest [112,113,114]. This is thought to occur through E4s ability to interact with cyclin B/Cdk1 complexes and to promote inhibitory phosphorylation of Cdk1 through the Wee1 kinase [114,115]. E1^E4 is the most abundantly expressed viral gene upon differentiation, occurring concomitantly with viral genome amplification due to activation of the late promoter [28]. In addition, HPV16 E4 protein stability is increased upon phosphorylation by ERK1/2, leading to high levels of E4 protein in differentiating cells [116]. E1^E4 expression has been shown to be necessary for efficient productive replication of HPV16, HPV18 and HPV31, but not for low-risk HPV11 [30,31,32,106]. Studies using normal immortalized keratinocytes containing HPV16 E4 mutants that no longer induce G2 arrest exhibit decreased viral genome amplification and L1 gene expression upon differentiation in methylcellulose, as well as in organotypic raft cultures [29]. These studies indicate that the G2 arrest function of E4 contributes to providing a replication-competent environment. The accumulation of E4 in G2 arrested cells may foster productive replication by enhancing the accumulation of E1 in the nucleus, possibly through activation of MAPK pathways that activate E1’s nuclear localization sequence [29,117,118]. E4 has been proposed to induce G2 arrest to counteract E7-induced proliferation in order to establish an environment that allows for rapid amplification of viral genomes. It is possible that E7 initiates G2 arrest following cell cycle re-entry through activation of ATM and ATR, but increased E4 protein levels sustain G2 arrest, providing an environment conducive to productive viral replication. Productive replication in a G2 arrested environment is postulated to allow HPV to avoid competition with host DNA synthesis and appropriate necessary cellular factors for amplification of its genomes. E2 may contribute to this process through interaction with the cellular replication protein ORC2 (origin recognition complex), which promotes assembly of pre-replication (pre-RC) complexes on mammalian origins. Overexpression of HPV31 or HPV16 E2 decreases ORC2 occupancy at mammalian origins [119], raising the possibility that increased levels of E2 upon differentiation may serve to restrict pre-RC assembly at cellular origins that could compete with HPV for access to host replication machinery. This is important considering that increasing evidence supports a role for homologous recombination (HR) DNA repair pathways in the amplification of HPV genomes (discussed below) [25,120]. HR activity is restricted to the S- and G2-phases of the cell cycle [121]. By productively replicating post-cellular DNA synthesis in a G2 arrested environment, HPV has unfettered access to DNA repair factors, as well as other cellular factors, that are necessary for viral DNA synthesis.

## 7. Use of DNA Damage Response Pathways for Productive Replication

**Figure 4:**
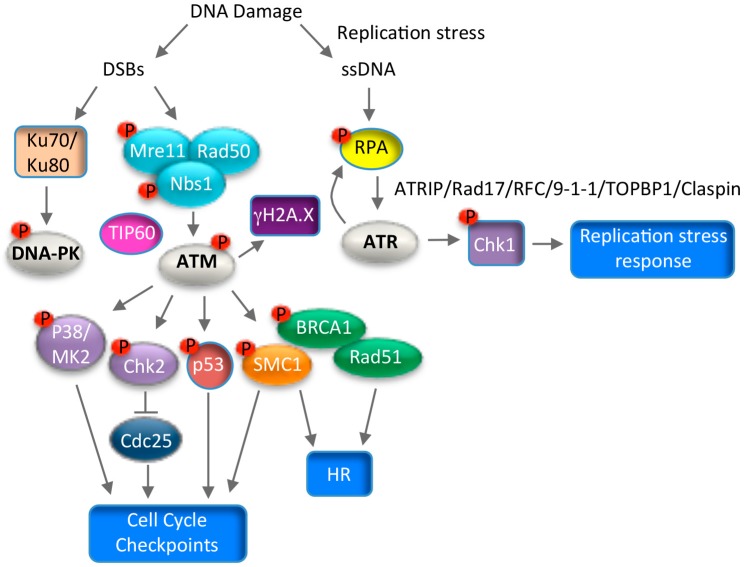
Schematic of the Ataxia-Telangiectasia Mutated (ATM), DNA-dependent Protein Kinase (DNA-PK), and ATM and Rad3-related (ATR) DNA damage response pathways. ATM and DNA-PK are activated in response to double strand DNA breaks (DSBs). ATM facilitates DNA repair through high-fidelity homologous recombination (HR), however, DNA-PK promotes repair through error-prone non-homologous end joining (NHEJ). DNA-PK is activated by the DNA damage sensor complex of Ku70/Ku80, while ATM is activated by the DNA damage sensor complex MRN (Mre11, Rad50, Nbs1) and the TIP60 acetyltransferase. ATM phosphorylates numerous downstream effectors, including H2A.X (gH2AX), Chk2, p38, p53, SMC1 and Breast Cancer Gene 1 (BRCA1) to induce cell cycle arrest and facilitate DNA repair, or to promote apoptosis in the case of severe DNA damage. ATR is activated by single-stranded DNA (ssDNA) generated by replication stress or the resection of DSBs. ssDNA is protected by the tripartite complex RPA, which promotes ATR activation through recruitment of ATRIP, a critical ATR regulator. The Rad17/RFC complex also binds to RPA-coated ssDNA and loads the 9-1-1 complex (Rad9-Hus1-Rad1). 9-1-1 recruits TOPBP1, which is necessary for ATR activation. Claspin mediates the activation of Chk1 by ATR, leading to the replication stress response. T bars indicates inhibition. Arrows indicate activation.

Numerous studies over the past several years have provided evidence to support a role for the DNA damage response (DDR) in productive replication of high-risk alpha HPV types [25]. The DDR is a complex series of signaling events that act to coordinate the cell cycle with DNA repair. There are three main kinases activated in response to DNA damage; ATM, ATR and DNA-PK (DNA-dependent Protein Kinase), all of which belong to the PIK-like kinase (Phosphatidyl inositol 3’ kinase) family of serine/threonine kinases [122]. ATM and DNA-PK respond primarily to double-strand DNA breaks (DSBs) and promote repair through high fidelity homologous recombination (HR), or error prone non-homologous end joining (NHEJ), respectively [121] (Figure 4). In contrast, ATR facilitates repair of single-strand DNA that is generated in response to replication stress, or during the processing of DSBs [123] (Figure 4). However, due to the complexity of DNA repair, there is considerable cross-talk between these pathways to maintain genomic integrity. HPV requires activation of the ATM and ATR response pathways for productive viral replication, however whether the DNA-PK pathway also contributes to viral replication is not yet known. Activation of the DDR provides HPV access to the necessary repair factors that play a direct role in viral DNA synthesis. In addition, increasing evidence suggests that HPV utilizes these pathways to establish a G2 arrested environment that is amenable to recombination-directed amplification of viral genomes.

### 7.1. ATM Signaling and Productive Viral Replication

**Figure 5:**
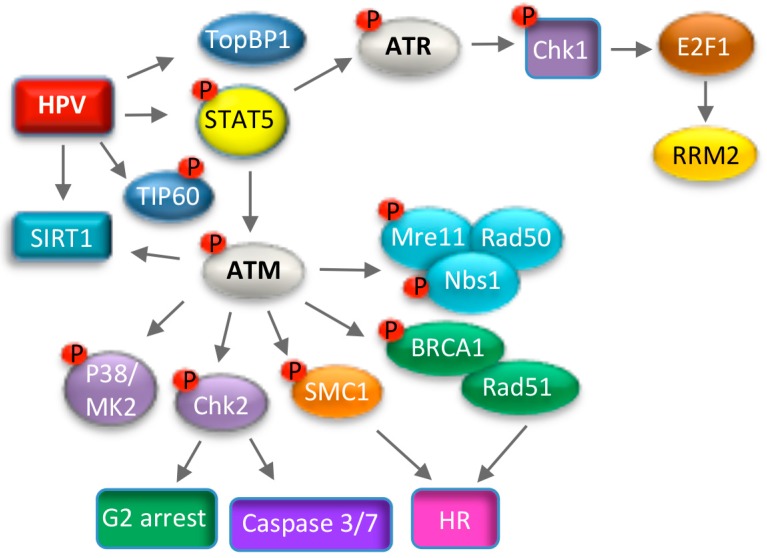
Modulation of the ATM and ATR DNA damage response pathways to promote productive viral replication. HPV-induced activation of ATM requires the STAT5 immune regulator, as well as TIP60, but not the MRN complex. Downstream effectors of ATM required for productive viral replication include the MRN complex, p38/MK2, Chk2, as well as factors involved in homologous recombination repair (Rad51, BRCA1, SMC1). HPV may utilize ATM activity to promote G2 arrest upon differentiation through activities of Chk2, as well as to direct repair to HR on viral genomes through epigenetic modifications and the recruitment of homologous recombination (HR) repair factors. ATR activation in HPV positive cells likely occurs through E7-induced replication stress and requires a STAT5-directed increase in TOPBP1. ATR/Chk1 activation leads to increased levels of E2F1, which drives expression of RRM2, resulting in increased dNTP pools to facilitate productive viral replication.

In response to DSBs, ATM is conically activated by the MRN (Mre11, Rad50, Nbs1) complex, which serves as a sensor of DNA damage, and by acetylation via the TIP60 acetyltransferase [122,124,125,126] (Figure 4). ATM then phosphorylates numerous downstream targets, including the histone variant H2A.X (histone 2A variant X), which initiates repair factor recruitment to sites of DNA damage in a highly ordered fashion [127]. ATM elicits its effects on cell cycle arrest and DNA repair through the activation of numerous kinases, including Chk2 and p38MAPK [122,128] (Figure 4). Chk2 phosphorylates many downstream effectors, including repair factors such as BRCA1 (Breast Cancer Gene 1), p53, and the Cdc25c family of phosphatases to mediate G2/M arrest [129]. p38MAPK (Mitogen Activated Protein Kinase) signaling is independent of Chk2 and induces the DDR through phosphorylation of MK2 (MAPK-activated protein kinase 2), which in turn phosphorylates downstream substrates to induce G2/M arrest [128]. The ATM effector SMC1 constitutes a third arm of the DDR, which along with Nbs1 induces cell cycle arrest and DNA repair [130,131] (Figure 4). A seminal study by the Laimins lab demonstrated that ATM is constitutively active in high-risk HPV31 positive cells [100], and is characterized by the phosphorylation of multiple downstream targets, including H2A.X, Chk2, Nbs1, BRCA1, SMC1, p38MAPK and MK2 [100,132,133] (Figure 5). Subsequent studies demonstrated similar findings for HPV16 and HPV18 [104,134]. Inactivation of the MRN complex in HPV31 positive cells does not abrogate ATM activation [135], suggesting that HPV utilizes a non-canonical mechanism to induce the ATM DDR necessary for productive viral replication. Intriguingly, activation of the ATM pathway is specifically required for productive replication of HPV31 upon differentiation, with inhibition of ATM activity having no effect on episomal maintenance in undifferentiated cells [100]. Similar results were observed for the ATM effector Chk2 [100]. In addition to inactivation of Cdc25c, Chk2 activity is also necessary in differentiating HPV31 positive keratinocytes for activation of caspase-3/7, which is required for cleavage of the E1 viral helicase and viral genome amplification [100,136]. Interestingly, in contrast to Chk2, activation of the p38/MK2 axis of the ATM DDR is induced only upon differentiation [132]. The p38/MK2 complex is also necessary for productive replication of HPV31, though the downstream targets of this complex that drive viral DNA synthesis have not been defined [132].

Multiple ATM signaling components are recruited to productively replicating viral genomes, including ATM, γH2A.X, Chk2, 53BP1, MRN, Rad51 and BRCA1, suggesting a direct role for DNA repair mechanisms in viral DNA synthesis [135,137,138,139]. Indeed, along with ATM, several of these factors, including the MRN complex, Rad51 and BRCA1, are necessary for DNA repair through homologous recombination (HR), and importantly, are also required for productive replication of HPV31 [101,122,135]. HR is a relatively error-free process, and HPV may preferentially use this method of repair to maintain the integrity of viral DNA during amplification. Structures consistent with recombination have been observed during productive replication of HPV31 and HPV16 that are not detected during maintenance replication in undifferentiated cells [140]. These observations suggest that amplification of viral genomes upon differentiation occurs in a distinct manner that may require ATM-driven HR. Initiation of HR requires resection of DSBs, which requires ATM activity, as well as BRCA1 and the MRN resection complex [121]. Resection is required for loading the Rad51 recombinase onto DNA, which then facilitates strand invasion into homologous sequences [122]. Rad51 binding to HPV31 DNA increases upon differentiation, and inhibition of Rad51’s DNA binding ability blocks productive viral replication, suggesting that viral DNA resection is necessary for amplification of viral genomes [101]. In support of this, Anacker et al. demonstrated that the MRN complex is required for Rad51 localization to HPV31 replication foci, and that Mre11’s nuclease activity is necessary for productive viral replication [135]. Recent studies have shown that SMC1 is also required for productive replication of HPV31 [133]. SMC1 is a member of the sister chromatid cohesion complex that is important for chromosome segregation during mitosis [141]. The role of SMC1 in productive viral replication is not clear, but SMC1 is recruited to the viral genome in a complex with CTCF insulator proteins [133]. SMC1 is postulated to promote HR by maintaining the close proximity of sister chromatids at DSBs [131], and may serve a similar role on HPV genomes to facilitate recombination-dependent replication.

In the context of the complete HPV31 genome, ATM activation occurs in a manner dependent on E7’s Rb binding domain [142]. Expression of HPV18 E7 alone in organotypic raft cultures results in activation of ATM, Chk2 and Chk1 in the suprabasal layers, offering support that E7 contributes to productive viral replication through eliciting ATM activation in differentiating cells [104]. HPV31 E7 regulates the activation of ATM through STAT5, an immune regulator that is required for productive viral replication [143] (Figure 5). How STAT5 leads to ATM activation is currently unclear, but may involve STAT5-dependent activation of TIP60 [144]. In addition to ATM activation, HPV31 E7 contributes to productive viral replication by increasing the protein half-life of several DNA repair factors that are required for productive replication (e.g., ATM, Chk2, Chk1, Mre11, Rad50, Nbs1, Rad51 and BRCA1), ensuring high levels for efficient viral DNA synthesis [142]. Expression of the E1 viral helicase alone from high-risk and low-risk HPV types is sufficient to induce ATM activation, which may occur through the induction of DSBs due to E1’s ability to non-specifically bind and unwind cellular DNA [134,145,146]. In the presence of E2, E1 is recruited to the viral origin of replication, along with multiple components of the ATM and ATR pathway [134,145,147]. How ATM activity is regulated by E7 versus E1 during the viral life cycle remains to be determined. In addition, whether activation of the ATM DDR occurs in the context of low-risk HPV infection, and if this response is required for productive replication is currently unknown. In contrast to the Alpha high-risk HPV types, beta HPV E6 and E7 proteins reduce expression of ATM and ATR, as well as the HR factors Rad51 and BRCA2, in turn delaying repair foci formation in response to UV exposure [148]. Whether inactivation of the DDR is necessary for the life cycle of beta HPVs is not yet known due to the lack of experimental systems to study replication.

The recruitment of DNA repair factors to sites of DNA damage requires alterations in chromatin structure orchestrated through ATP-dependent remodeling complexes and post-translational modifications of histones (e.g., acetylation, ubiquitylation, phosphorylation, methylation) [149,150]. ATM-induced phosphorylation of H2A.X (γH2A.X) is one of the key effectors in modulating chromatin dynamics in response to DSBs [127]. γH2A.X initiates the assembly of repair factors at DNA lesions in a highly regulated manner, including HR factors (MRN, Brca1 and Rad51) [151]. γH2A.X is bound to HPV31 DNA and binding increases during productive viral replication, suggesting that γH2A.X may serve to assemble HR repair factors at viral replication sites [138,152]. The DDR-associated histone deacetylase SIRT1 and the acetyltransferase TIP60 have also been linked to productive viral replication. SIRT1 channels repair to HR by recruiting Nbs1 and Rad51 to damaged DNA in an ATM- and γH2AX-dependent manner [153]. Interestingly, SIRT1 binds to HPV31 DNA and is necessary for productive viral replication, which may be mediated through the recruitment of Nbs1 and Rad51 to viral replication foci [139]. TIP60 is upregulated in HPV31 positive keratinocytes and is also necessary for productive viral replication [144]. While this presumably is due to TIP60’s role in ATM activation, TIP60 can also influence repair to the HR pathway through the acetylation of histone H4 and attenuation of 53BP1 binding, which promotes repair through NHEJ [154]. SIRT1 and TIP60 may modify viral chromatin to ensure the recruitment of HR factors to productively replicating viral genomes. How the HPV life cycle may be epigenetically regulated through ATM activity is an interesting area of investigation.

### 7.2. ATR Signaling and Productive Viral Replication

Replication stress results in formation of single strand DNA (ssDNA) at stalled replication forks that activates the ATR kinase [155]. ATR and its downstream target Chk1 protect stalled replication forks and prevent excessive origin firing, maintaining genome integrity. High-risk HPV positive cells exhibit constitutive activation of the ATR pathway, indicating that replication stress is a chronic problem that HPV has to contend with [100,110,111]. Unscheduled cell cycle entry induced by high-risk HPV E6 and E7 proteins results in replication stress due to a disconnect between activation of cellular DNA synthesis and the availability of supplies required for replication [156,157]. This is thought to occur through E7’s ability to target Rb for degradation. In support of this, mutation of E7’s Rb binding domain in the context of the HPV31 genome prevents ATR signaling [142]. ATR activation requires recruitment to RPA-coated ssDNA by its regulator ATRIP [158] (Figure 4). ssDNA-RPA also recruits the RFC/Rad17 complex, which facilitates loading of the 9-1-1 complex at stalled replication forks [159]. The 9-1-1 complex then recruits TOPBP1 to activate ATR’s kinase activity [160]. Intriguingly, HPV31 E7 ensures that infected cells can sufficiently respond to replication stress through ATR activation by increasing the levels of TOPBP1 in a STAT5-dependent manner [110] (Figure 5). Although E1 of high-risk and low-risk types can also independently activate ATR, it is unclear if this results from non-specific binding and unwinding of cellular DNA, or if increased E1 activity on viral DNA during productive viral replication results in in replication stress [134,145].

Inhibition of ATR, as well as its downstream target Chk1, blocks productive replication of HPV31, and also decreases HPV31 and HPV16 copy number in undifferentiated cells [110,111,161]. In response to replication stress, ATR phosphorylates RPA on Ser33 [162]. pRPA Ser33 localizes to HPV31 replication foci, suggesting that viral genomes are subject to replication stress during productive replication [138]. Activation of the ATR/Chk1 pathway may be important in repairing stalled forks that occur during amplification of viral genomes. Upon replication stress, activation of the ATR/Chk1 pathway is instrumental in maintaining E2F signaling, ensuring the expression of cellular genes that facilitate DNA repair and cell survival [163]. This is particularly important in cancer cells, which typically exhibit high levels of replication stress [164,165,166]. Recent studies from our lab demonstrated that HPV31 utilizes the ATR/Chk1/E2F1 arm of the DDR to increase levels of RRM2, the small subunit of the ribonucleotide reductase complex, in an E7-dependent manner [111] (Figure 5). RRM2, along with the large subunit RRM1, is necessary for the conversion of ribonucleotides to deoxyribonucleotides, providing dNTPs for replication, DNA repair and survival [167]. Knockdown of RRM2 reduced dNTP pools in differentiating HPV31 positive cells and blocked productive replication [111]. These studies indicate E7 induced cell-cycle re-entry upon differentiation results in replication stress that activates the ATR/Chk1 pathway to maintain E2F signaling. Importantly, these studies demonstrate that HPV exploits the ATR DNA damage response to ensure an adequate supply of dNTPs for productive replication, providing a replication competent environment in cells that are no longer dividing. Understanding the full extent of the ATR pathway throughout the viral life cycle is an important area of future investigation.

### 7.3. Consequences of Utilizing the DNA Damage Response for Replication

Studies have shown that HPV replication foci tend to form near common fragile sites, which are regions of the cellular genome that are prone to replication stress and recruit DNA repair factors to maintain genomic stability [168,169]. HPV may preferentially replicate adjacent to fragile sites to readily have access to DNA repair factors to facilitate recombination-directed replication. Interestingly, in cancers associated with oncogenic HPV types, viral DNA is often found integrated into host DNA at common fragile sites [170,171,172,173]. Integration is a dead-end for virus production and almost always results in increased expression of the E6 and E7 oncogenes [174]. Deregulated E6/E7 expression leads to a proliferative advantage and the clonal outgrowth of cells containing integrated viral DNA. While replicating near areas of cellular replication stress may be beneficial to viral persistence and productive viral replication, the close association of HPV replication foci with areas of the cellular DNA damage may increase the chance of accidental integration of the viral genome, and may explain the tendency for HPV to integrate into common fragile sites of host DNA [175]. Furthermore, recent studies from the Galloway lab demonstrated that high-risk E6 and E7 proteins attenuate the repair of cellular DSBs through the HR pathway. While this likely ensures HR factors are available for viral replication, the presence of persistent, unrepaired DNA breaks increases the opportunity for viral genome integration [176]. These integration events, in turn, may contribute to HPV oncogenesis through E6/E7-mediated genomic instability.

## 8. Conclusions

In order to provide a replication-competent environment, HPVs co-opt particular host cell pathways and interactions that regulate epithelial differentiation and cellular proliferation, as well facilitate repair of damaged DNA. Temporal regulation of viral gene expression is necessary to restrict high levels of viral gene expression, replication and virion production to the uppermost layers of the epithelium, protecting HPV-infected cells from detection by the immune response. This is achieved through differential usage of promoters and polyadenylation sites, as well as alternative splicing. In addition, E6 and E7 play critical roles in modulating innate immune responses to facilitate viral persistence and promote viral replication [27]. Cooperation between the activities of E6, E7, E1, E2, E4 and E5 upon differentiation allows HPV to establish an environment supportive of productive replication in non-dividing cells. Our understanding of how HPVs regulate the productive phase of the viral life cycle has increased dramatically over the past several years, particularly regarding how high-risk HPVs activate and utilize DNA repair pathways to amplify viral genomes. However, much remains to be learned regarding how alpha HPVs manipulate cellular pathways to facilitate viral replication, and in turn, how hijacking these pathways may affect the integrity of the cellular genome. Further understanding of the mechanisms by which HPV establishes a replication-competent environment throughout the viral life cycle is important to identify novel cellular targets that could be exploited therapeutically for the treatment of HPV-associated diseases.
